# Measuring executive dysfunction in Parkinson’s disease: Reliability and validity of the Spanish version of Frontal Assessment Battery (FAB-E)

**DOI:** 10.1371/journal.pone.0207698

**Published:** 2018-11-19

**Authors:** Miriam Hurtado-Pomares, María Carmen Terol-Cantero, Alicia Sánchez-Pérez, Carlos Leiva-Santana, Paula Peral-Gómez, Desirée Valera-Gran, Eva María Navarrete-Muñoz

**Affiliations:** 1 Department of Pathology and Surgery, Miguel Hernandez University, San Juan de Alicante, Spain; 2 Department of Health Psychology, Miguel Hernandez University, San Juan de Alicante, Spain; 3 Department of Clinical Medicine, Miguel Hernandez University, San Juan de Alicante, Spain; 4 Department of Neurology, General University Hospital of Alicante, Alicante, Spain; 5 Institute for Health and Biomedical Research (ISABIAL-FISABIO Foundation), Alicante, Spain; 6 Department of Public Health, History of Medicine and Gynecology, Miguel Hernandez University, San Juan de Alicante, Spain; 7 CIBER de Epidemiología y Salud Pública (CIBERESP), Institute of Health Carlos III (ISCIII), Madrid, Spain; Universidade Federal do Rio de Janeiro, BRAZIL

## Abstract

**Background:**

Deficits in executive functions (EFs) are frequently detected in patients with Parkinson’s disease (PD). The Frontal Assessment Battery (FAB) is a screening test for assessing EFs although it has not been so far adapted and validated in Spain. We evaluated the reliability and validity of the Spanish version of the FAB (FAB-E) in PD patients.

**Materials and methods:**

Our study included 54 healthy subjects and 67 PD patients. Cognitive assessment of participants was conducted using the FAB-E, Mini-Mental State Examination (MMSE), Trail Making Test (TMT), Revised-Barcelona Test (RBT) and Executive Interview (EXIT-25). Internal consistency, intra- and test-retest reliabilities, concurrent and discriminant validity of the FAB-E were examined. To evaluate the influence of cognitive dysfunction in PD on the performance of the FAB-E, we also classified the PD patients into groups according to their cognitive status as measured by the MMSE using published criteria to identify cognitive deficits in PD.

**Results:**

The FAB-E showed good internal consistency (α = 0.751). The intraclass correlation coefficients (ranging from 0.559 to 0.891) and Spearman correlations (from 0.494 to 0.864) of the FAB-E subtests indicated a good-strong reliability. The total and subtest scores generally showed a good concurrent validity, except for the prehension behaviour item of the FAB-E and the Interference and Go/no-go tasks of the EXIT-25 that presented low estimates. Excluding the prehension behaviour subtest, the performance of the FAB-E was higher in the control group than in PD patients. Cognitive dysfunction in PD patients also indicated significant poorer FAB-E scores excepting the motor and prehension behaviour subtests. Discriminant analysis determined a cut-off of 14.5 was optimal to differentiate healthy subjects from PD patients. Moreover, a cut-off <12.5 allocated satisfactorily those PD patients with cognitive impairment (MMSE<26) and scores <11.5 classified suitably those PD patients with dementia (MMSE<24).

**Conclusion:**

The FAB-E is an accurate tool for evaluating EFs in patients with PD and can provide useful information for distinguishing PD patients with and without cognitive dysfunction at a bedside assessment.

## Introduction

Parkinson’s disease (PD) is a progressive, disabling, neurodegenerative disorder which is traditionally considered as a motor system disorder characterized by shaking, rigidity, bradykinesia and postural instability. However, the non-motor symptoms (e.g. cognitive impairment (CI), depression, psychiatric, autonomic and sleeping disorders, apathy and pain) of PD are the main causes of morbidity requiring institutionalization or hospitalization in the advanced stages of the disease [1]. Although PD does not necessarily imply dementia and patients can maintain their abilities to normally perform their daily activities, in the early stages of the disease, due to frontal lobe and basal ganglia circuitry dysfunctions, cognitive deficits can be observed in patients, predominantly affecting visual-spatial functions, memory and executive functions (EFs)[2]. Particularly, they often present difficulties for tasks that require planning, problem-solving, cognitive flexibility, generating strategies, sequencing and abstract verbal reasoning that lead to a negative impact on daily activities [2,3]. However, these non-motor symptoms remain largely unrecognized and, therefore, untreated [4]. In respect to this, similar to that proposed for Alzheimer’s disease, new research perspectives are focusing on the need of a neuropsychological assessment in the early or prodromal stage of PD to study initial cognitive decline [5], which will help greatly to design early therapeutic interventions to improve long-term outcomes.

Deficits in EFs are commonly manifestations detected in PD that may induce detrimental consequences to an equal or greater extent than the limitations resultant from the progressive impairing effects of the motor functions [6]. In fact, research focused on cognitive dysfunction in PD suggest that deficits in EFs seem to be the cognitive symptoms that contribute greatly to the impairment caused by the disease [3]. Hence, it is important to have accurate instruments to assess executive dysfunctions that may help to understand the progress of the disease, as well as visualizing the impact and needs of those affected by PD.

There is a wide range of tests that evaluate impairment of EFs, although the vast majority of them are very extensive and take a long time to administer, and examiners need a lot of training and experience. Moreover, motor difficulties can represent an important confounding effect in most of these tests that depend on the time to complete a task (e.g. Trail-Making Test). On the contrary, the Frontal Assessment Battery (FAB) [7] is a brief, simple and clinically useful screening tool to assess EFs [8] that takes approximately 10 minutes. It is composed of 6 sub-scales evaluating cognitive and behavioral domains involved in the different neural networks, which not only permits profiling an executive dysfunction, but also exploring related sub-syndromes [9]. Different studies have shown that the FAB has excellent psychometric properties of reliability and validity for its application in different countries and for several pathologies, including PD [10–16]. As far as we know, the FAB has not been previously adapted and validated in Spain. For the present article, we aimed to evaluate the psychometric properties of validity and reliability of the Spanish version of the FAB (FAB-E) in patients with PD.

## Materials and methods

### Ethics statements

The present study was approved by the ethics committees of the Miguel Hernández University (DPC-MHP-001-11) and the General University Hospital of Alicante (PI2011/52). Before enrolment in the study, all participants provided written informed consent.

### Participants

The study sample consisted of a control group of 54 healthy subjects and a patient group formed of 67 individuals with PD. The diagnostic criteria for PD were based on the United Kingdom Parkinson’s Disease Society (UKPDS) Brain Bank criteria[17]. All the patients included were diagnosed as idiopathic PD and a likely diagnosis of vascular Parkinsonism was discarded based on the poor response of patient to Parkinson’s medications. Moreover, the patients with presence of cerebrovascular disease were not included in the study. The PD patients were recruited from the Parkinson’s Association of Alicante and the Neurology Unit of General University Hospital of Alicante, and the participants for the control group were enlisted among the relatives of PD patients and elderly people enrolled in the “Aulas de la Experiencia” (Third Age education program) at Miguel Hernández University. The inclusion criteria of the study were that candidates were native Spanish speakers, not illiterate, and 50 years or older, since the incidence of PD in patients less than 50 years is very low[18]. All the participants were excluded if they had a history of the following medical conditions: central nervous system disease with a neurological alteration (acquired brain damage, epilepsy, traumatic brain injury, multiple sclerosis and other movement disorders); visual or hearing impairment; metabolic disorders (diabetes mellitus, hypothyroidism); serious psychiatric illness (depression, psychosis, schizophrenia); and current or past history of alcohol or drug abuse.

Information on socio-demographic features of all participants was also collected and clinical data about main diagnosis was obtained from medical reports of the PD patients.

### Instruments

#### Frontal Assessment Battery

Before initiating this study, we developed the Spanish version of the FAB (FAB-E) performing a cross-cultural adaptation of the original English version of the FAB into Spanish, following a thorough translation/back-translation methodology[19]. After considering the recommendations and suggestions by the author of the original test, Bruno Dubois[7], it was pilot-tested in 19 healthy individuals in order to ascertain the final version of the test.

The FAB consists of 6 subtests that explore each of the processes controlled by the frontal lobes: 1) similarities (abstract reasoning/conceptualization); 2) lexical fluency (mental flexibility [i.e. self-organization, strategy and change]); 3) motor series (programming and motor planning); 4) conflicting instructions (sensitivity to interference); 5) Go-no-go test (inhibitory control and impulsiveness); and 6) prehension behavior (ability to inhibit a response to sensorial stimulation [i.e. environmental autonomy]). Higher scores of the test imply a better performance, and the total maximum score that can be obtained in the FAB is 18. The scoring is calculated by adding up the points for each test, which ranges from 0 to 3.

#### Other neuropsychological instruments

General cognitive functioning was assessed using the Spanish version of the MMSE[20] which examines cognitive abilities of temporal and spatial orientation, memory, registration, attention, calculation, recall, constructive language and praxis. For the examination of executive functioning, given that a “gold standard” to determine the existence and severity of a frontal lobe syndrome[3,7] is not available, we used several neuropsychological tests of EFs. Thus, we included the following commonly used tests, adapted and validated for Spanish population: the Trail Making Test (TMT A and B)[21], which evaluates EFs, i.e. sustained visual attention, sequencing, mental flexibility, visual tracking and graphomotor skill; the Revised Barcelona Test (RBT)[22], which assesses orientation, attention, language, reading, praxis, gnosis, memory, abstraction and EFs, and only using for the present study the subtest 12 (Categorical evocation), subtest 23 (Position sequences, i.e. right and left hands) and subtest 39 (Verbal abstractions, i.e. similarity/abstraction); and the Executive Interview (EXIT-25)[23], which evaluates EFs encompassing a set of several domains: perseveration, imitation, echopraxia, echolalia, intrusions, frontal liberation signs, lack of spontaneity, disinhibition, and utilization behaviour. For the validation task, we used the item 7 (Interference task), item 15 (Go-no-go task), and item 19 (Prehension task).

All the neuropsychological testing in the PD patients was performed in the “on” medication state to minimize potentially confounding motor effects.

#### Statistical analysis

Descriptive analyses were made to compare socio-demographic characteristics and neuropsychological examination according to the different groups of participants. The normal distribution of the data was checked by Kolomogorov-Smirnov test. Due to the continuous variables in our sample were not normally distributed, they were displayed as median and interquartile range (IR). Thus, the reliability and validity measures were performed using nonparametric statistics. To determine the consistency of the scores of the FAB-E administered in two separate occasions over a period between two and four weeks, intra-rater and test-retest reliabilities were analyzed in terms of intraclass correlation coefficient (ICC) and Spearman correlation coefficient, respectively. A good ICC was considered a coefficient of 0.75 or higher [24], and a value of 0.50 or higher indicated a strong Spearman correlation[25]. Moreover, a good internal consistency as measured by the Cronbach’s alpha was deemed a value of 0.70 or higher [26]. Concurrent validity between the MMSE, TMT (A and B), RBT (subtests 12, 23 and 39), and EXIT-25 (items 7, 5 and 19) and the FAB-E subtest and total scores was determined by Spearman correlation coefficients.

To evaluate whether the cognitive symptoms caused by PD provided further information on the performance of the FAB-E, we divided the PD patients into groups according to their cognitive status as measured by the MMSE and classified using published criteria to identify cognitive deficits in PD[27–29]. A MMSE score of 26 was used to classify PD patients into those without CI (PD-nonCI; MMSE≥26) and those with CI (PD-CI: MMSE<26). A MMSE score lower than 24 was used as cutoff to detect severe CI or probable dementia, dividing patients into those without dementia (PD-nonD: MMSE≥24) and those with dementia (PD-D: MMSE<24). Within-group comparisons were carried examining differences in the FAB-E scores.

We also assessed the discriminant validity by performing a forward stepwise discriminant function analysis to differentiate between groups, i.e. healthy subjects vs. PD patients, and within patient group (PD-nonCI vs. PD-CI, and PD-nonD vs. PD-D). To control the likely influence of age, it was included in the models as a covariate. The area under the ROC curve (AUC) was used to calculate the optimal total FAB-E cut-off point for detecting executive dysfunction among healthy participants and PD patients, and among those with and without CI or dementia within PD patients. To examine whether the findings of the ROC analysis varied by sociodemographic characteristics, we replicated the analysis of the specified subgroups within the strata of age, sex and educational level. However, we were not able to conduct this analysis in the PD-D subgroup due to the small size of sample (n = 18).

Statistical significance was established at a P value <0.05 and all the analyses were conducted with R statistical software version 3.3.3. (R Foundation for Statistical Computing, Vienna, Austria; http://www.r-project.org). For the ROC analyses, we also used pROC package of R statistical software [30].

## Results

### General characteristics of the sample

Sociodemographic characteristics of the participants as well as the total scores obtained in the MMSE and neuropsychological tests of EFs are displayed in Table 1. Overall, significant differences between healthy controls and PD patients were observed. PD patients were significantly older and had a higher proportion of men compared to healthy group. However, the distribution of the level of education did not differ significantly among groups. In patient group, the median duration of PD symptoms was 5 years (IR, 2–7 years). Regarding cognitive assessment, healthy subjects presented significantly the highest scores in the MMSE and all the items of the TMT and RBT, although the EXIT-25 scores did not show differences between both groups of participants.

**Table 1:** General characteristics of study participants (n = 121).

	Healthy subjects (n = 54)	PD patients (n = 67)	P^a^
Age, median (IR)	62 (60–67)	70 (64.5–77.5)	<0.001
Gender, n (%)			0.006
Women	37 (68.5)	29 (43.3)	
Men	17 (31.5)	38 (56.7)	
Level of education, n (%)			0.069
Primary studies or less	23 (42.6)	40 (59.7)	
Secondary studies or more	31 (57.4)	27 (40.3)	
Duration of PD symptoms, median (IR)	-	5 (2–7)	-
MMSE, median (IR)	28.5 (27.25–29)	26 (23–28.5)	<0.001
TMT			
TMT-A, median (IR)	43.9 (37.3–54.5)	72.2 (57–127.3)	<0.001
TMT-B, median (IR)	93.6 (73.3–123.1)	179.9 (111.5–300)	<0.001
RBT			
Item 12, median (IR)	26.5 (22.25–32)	21 (15–29)	0.002
Item 23 right hand, median (IR)	7 (7–8)	5 (4–7)	<0.001
Item 23 left hand, median (IR)	6 (6–7)	4 (2–6)	<0.001
Item 39, median (IR)	8 (7–9)	6 (3.5–8)	<0.001
EXIT-25			
Interference task, median (IR)	0 (0–0)	0 (0–0)	0.424
Go/no-go task, median (IR)	0 (0–0)	0 (0–1)	0.074
Prehension task, median (IR)	1 (0–2)	2 (0–2)	0.235

Abbreviations: SD, standard deviation; MMSE, Mini Mental State Examination; TMT, Trait Making Test; RBT, Revised-Barcelona Test; EXIT-25, Executive Interview; IR, interquartile range.

^a^P value from the Mann-Whitney U test (non-parametric continuous variables) and Fisher’s test (dichotomous categorical variables).

### Internal consistency, intra-rater and test-retest reliabilities

Table 2 displays the results of the internal consistency and the reliability measures of the FAB-E subtests among PD patients. The Cronbach’s alpha coefficient of the FAB-E items was 0.751, suggesting good internal consistency. Overall, the alpha reliability remained similar when an item was deleted from the test. Intra-rater reliability of the FAB-E subtests was moderately good or good, with estimates ranging from 0.559 (conflicting instructions item) to 0.891 (prehension behaviour item). The test-retest reliability indicated strong correlations with estimates ranging between 0.494 and 0.864 in the same FAB-E subtests as observed in the intra-rater coefficients.

**Table 2:** Internal consistency and reliability measures of the FAB-E scores among PD patients (n = 67).

	αª	ICC	r_s_
FAB-E items			
Similarities	0.736	0.619***	0.656***
Lexical fluency	0.696	0.749***	0.661***
Motor series	0.715	0.640***	0.641***
Conflicting instructions	0.676	0.559***	0.494***
Go-no go	0.689	0.666***	0.651***
Prehension behaviour	0.762	0.891***	0.864***

Abbreviations: ICC, intraclass correlation coefficient; r_s_, Spearman correlation coefficient.

ªCronbrach’s α if item is deleted.

* p<0.05

** p<0.01

***p<0.001

### Concurrent validity

Table 3 presents the results from the concurrent validity analysis, namely the Spearman correlations between the total and each item of the FAB-E and the other chosen neuropsychological tests in PD patients. The total and subtest scores of the FAB-E generally showed strong correlations for the MMSE and the tests of EFs, except for the prehension behaviour item of the FAB-E and the Interference and Go/no-go tasks of the EXIT-25 that globally presented low estimates for all the neuropsychological tests.

**Table 3:** Concurrent validity using Spearman correlations between the performance on the FAB-E and the other neuropsychological tests among Parkinson’ disease patients (n = 67).

	Total and subtest scores of the FAB-E						
	Total	Similarities	Lexical fluency	Motor series	Conflicting instructions	Go-no go	Prehension behaviour
MMSE	0.602***	0.516***	0.554***	0.285*	0.411***	0.331**	0.145
TMT							
TMT-A	-0.761***	-0.493***	-0.422***	-0.581***	-0.486***	-0.617***	-0.192
TMT-B	-0.744***	-0.473***	-0.423***	-0.537***	-0.376**	-0.536***	-0.115
RBT							
Item 12	0.554***	0.526***	0.734***	0.230*	0.262*	0.251*	0.123
Item 23 right hand	0.659***	0.543***	0.388**	0.480***	0.357**	0.456***	0.147
Item 23 left hand	0.661***	0.429***	0.360**	0.598***	0.396***	0.411***	0.237
Item 39	0.582***	0.575***	0.509***	0.227	0.437***	0.280*	0.171
EXIT-25							
Interference task	0.115	0.163	-0.035	0.203	-0.025	0.069	0.054
Go/no-go task	-0.172	-0.033	-0.083	-0.083	-0.157	-0.270*	-0.005
Prehension task	-0.380**	-0.311**	-0.294*	-0.125	-0.349***	-0.295*	0.151

Abbreviations: MMSE, Mini Mental State Examination; TMT, Trait Making Test; RBT, Revised-Barcelona Test; EXIT-25, Executive Interview.

* p<0.05

** p<0.01

***p<0.001.

### Comparison of the performance of the total and subtest FAB-E scores

Tables 4 and 5 display the results of the between-group (i.e. control group vs. PD patients) and within-group (i.e. PD-nonCI vs. PD–CI, and PD-nonD vs PD-D), respectively. With the exception of the Prehension behaviour item, statistically significant differences in all the FAB-E scores were observed between the control group (higher scores) and PD patients (lower scores). Among PD patients, the results showed that the degree of the cognitive dysfunction was proportional to the magnitude of executive deficits, thereby indicating statistically significant differences between PD groups except for the motor and prehension behaviour subtests of the FAB-E.

**Table 4:** Comparison of the performance of the FAB-E scores between control group and Parkinson’s disease patients (n = 121).

	Healthy subjects (n = 54)	PD patients (n = 67)	Pª
FAB-E items, median (IR)			
Similarities	2 (2–3)	1 (0.5–2)	<0.001
Lexical fluency	3 (2–3)	2 (2–3)	0.002
Motor series	3 (3–3)	3 (1–3)	<0.001
Conflicting instructions	3 (3–3)	2 (2–3)	<0.001
Go-no go	3 (3–3)	2 (1–3)	<0.001
Prehension behaviour	3 (3–3)	3 (3–3)	0.236
Total	16 (15–17)	13 (11–15)	<0.001

Abbreviations: PD, Parkinson’s disease; IR, interquartile range.

ªP value from Mann-Whitney U test.

**Table 5:** Comparison of the performance of the FAB-E scores between Parkinson’s disease patients with and without cognitive impairment, and with and without dementia (n = 67).

	PD-nonCI (n = 40)	PD-CI (n = 27)	P^a^	PD-nonD (n = 49)	PD-D (n = 18)	P^a^
FAB-E items, median (IR)						
Similarities	2 (1–2)	1 (0–1)	<0.001	2 (1–2)	0 (0–1)	<0.001
Lexical fluency	3 (2–3)	2 (0–2)	<0.001	3 (2–3)	1 (0–2)	<0.001
Motor series	3 (1–3)	2 (1–3)	0.103	3 (1–3)	2 (1–3)	0.529
Conflicting instructions	3 (2–3)	2 (1–3)	0.005	3 (2–3)	2 (0.25–3)	0.021
Go-no go	2 (2–3)	2 (2–3)	0.073	2 (2–3)	1 (0.25–2)	0.009
Prehension behaviour	3 (3–3)	3 (3–3)	0.654	3 (3–3)	3 (3–3)	0.278
Total	15 (12–16)	11 (7.5–13)	<0.001	14 (12–16)	9.5 (6.25–11.75)	<0.001

Abbreviations: PD-nonCI, Parkinson’s disease patients without cognitive impairment; PD-CI, Parkinson’s disease patients with cognitive impairment; PD-nonD, Parkinson’s disease patients without dementia; PD-D, Parkinson’s disease patients with dementia; IR, interquartile range.

^a^P value from Kruskal-Wallis rank sum test.

### Discriminant validity

The discriminant analysis adjusted for age showed that the FAB-E correctly identified 76.9% of the cases between healthy subjects and PD patients (r for canonical discriminant function (rCDF) was 0.511, Wilks’ lambda (λ) = 0.739, p<0.001), 73.1% of the cases between PD-nonCI and PD-CI patients (rCDF = 0.514, λ = 0.736, p<0.001), and 79.1% of the cases between PD patients with and without dementia (rCDF = 0.487, λ = 0.763, p<0.001). A stepwise discriminant analysis using the six FAB-E items as independent variables to discriminate between healthy subjects and PD patients showed that the similarities FAB-E subtest was the best item to correctly classify them (76.0% of patients, rCDF = 0.429, λ = 0.755, p<0.001), while the lexical fluency FAB-E subtest was the worst item (66.1% of patients, rCDF = 0.378, λ = 0.857, p<0.001). However, to distinguish PD patients without CI from those with CI or dementia, we observed that the lexical fluency FAB-E subset was the best item to allocate them (71.6% of patients, rCDF = 0.563, λ = 0.683, p<0.001; and 79.1% of patients, rCDF = 0.539, λ = 0.710, p<0.001, respectively). The FAB-E subtests with lower discriminant power among PD patients were Prehension behaviour (59.7% of the patients, rCDF = 0.225, λ = 0.949, p = 0.190) to differentiate those patients with CI, and the same FAB-E item (59.7% of the patients, rCDF = 0.226, λ = 0.949, p = 0.186) and Conflicting instructions subtest (59.7% of the patients, rCDF = 0.367, λ = 0.865, p = 0.010) to discriminate those patients with dementia.

**Fig 1:**
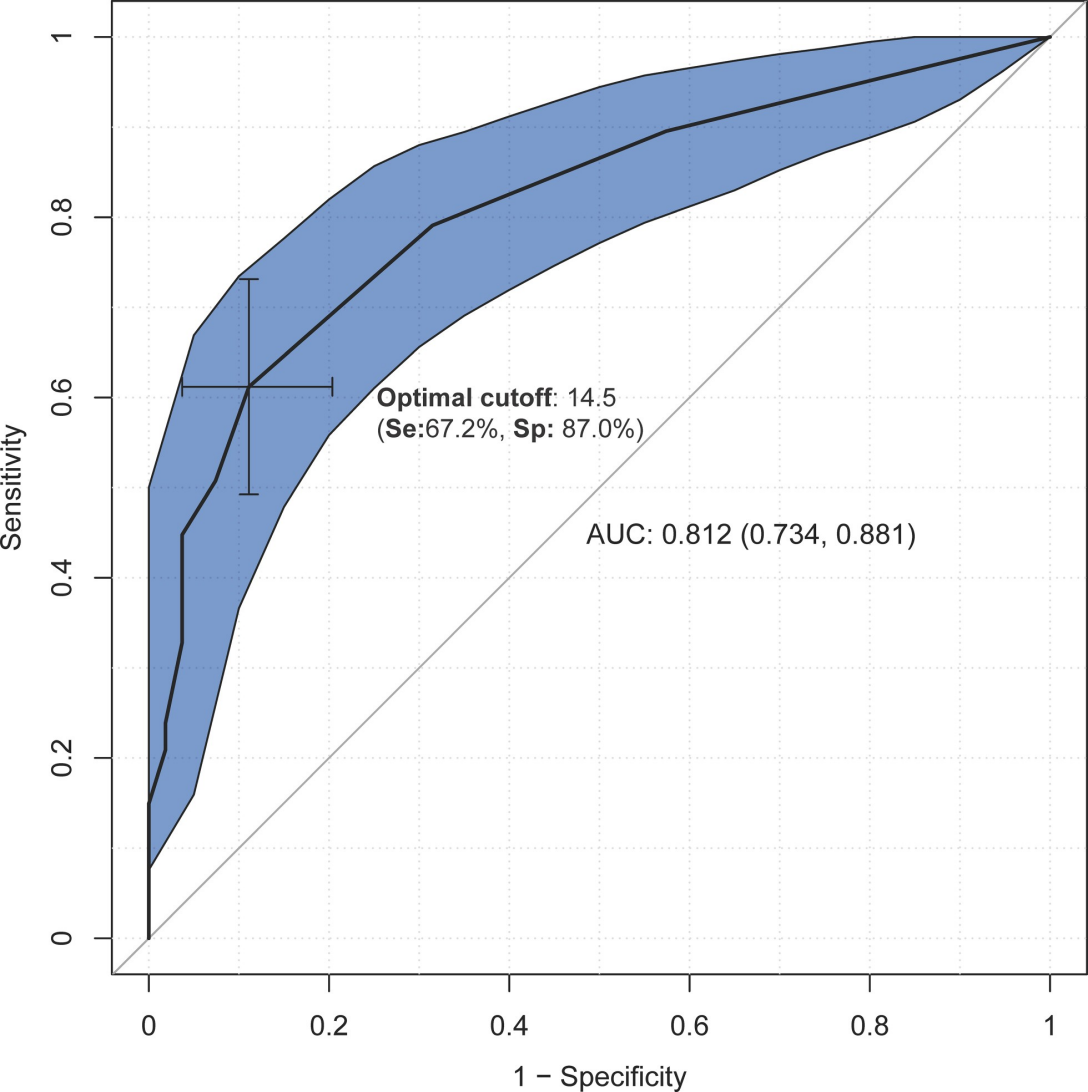
Discriminant power of the FAB-E among healthy subjects and Parkinson’s disease patients. Abbreviations: Se, sensitivity; Sp, specificity; AUC, area under the curve.

**Fig 2:**
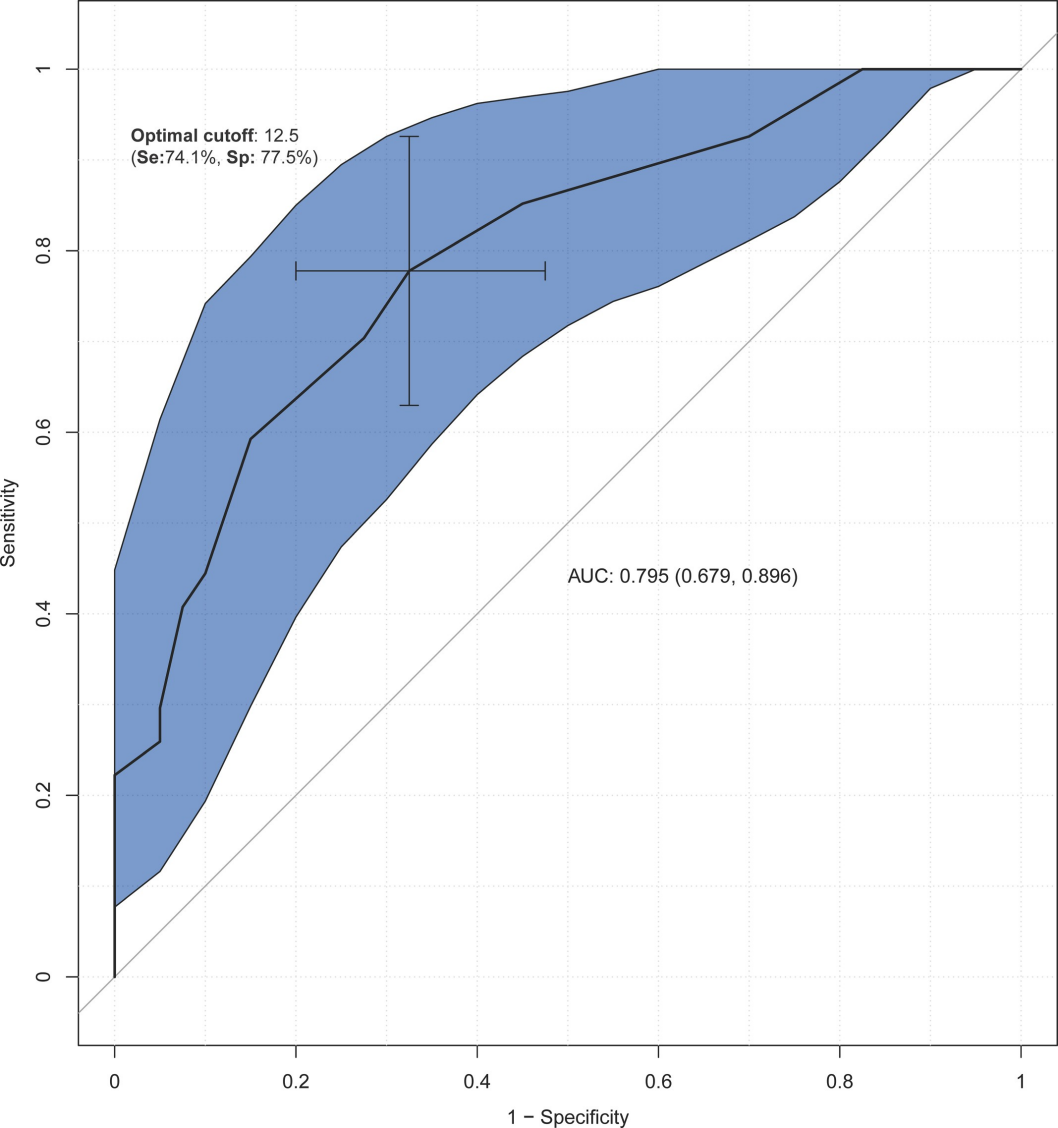
Discriminant power of the FAB-E among Parkinson’s disease patients with (MMSE<26) and without (MMSE≥26) cognitive impairment. Abbreviations: Se, sensitivity; Sp, specificity; AUC, area under the curve.

**Fig 3:**
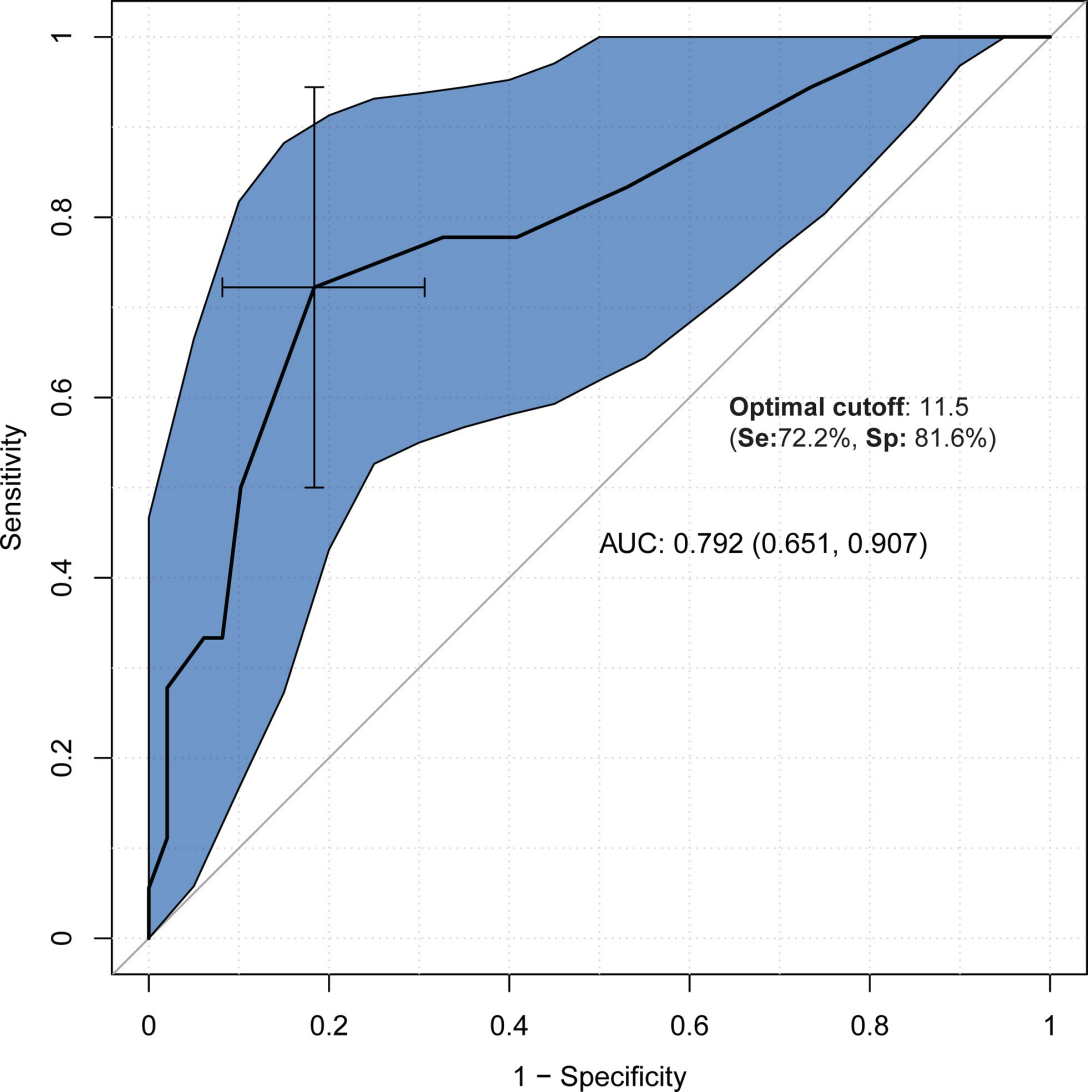
Discriminant power of the FAB-E among Parkinson’s disease patients with (MMSE<24) and without (MMSE≥24) dementia. Abbreviations: Se, sensitivity; Sp, specificity; AUC, area under the curve.

Figs 1–3 show the results of the ROC analysis. Compared to healthy subjects, the cut-off point of 14.5 on the total FAB-E was optimal for detecting executive dysfunction in PD with a sensitivity of 67.2% and specificity of 87.0%. The AUC of the FAB-E for PD was 0.812, suggesting a good diagnostic accuracy. The optimal cut-off point for identifying the presence of CI or dementia among PD patients using the FAB-E was 12.5 and 11.5 respectively, which also indicated a good diagnostic accuracy (AUC = 0.795 for CI and AUC = 0.792 for dementia), and yielded a sensitivity of 74.1% and 72.2%, and a specificity of 77.5% and 81.6% respectively.

Table 6 shows the results of the ROC analysis when we stratified for different subgroups of age, sex, and educational level. We did not observe substantial changes for the detection of executive dysfunction between healthy controls and PD patients (i.e. Control vs PD), except for those less than 67 years old (a cutoff of 13.5) and those with secondary studies or higher (a cutoff of 15.5). In PD patients, a cutoff of 11.5 was estimated as optimal within men and those with primary studies or less to distinguish between PD-nonCI and PD-CI. By contrast, a cutoff of 15.5 was estimated as optimal within those less than 67. All the AUC values suggested a good diagnostic accuracy (i.e. AUC ≥0.7), except for participants aged less than 67.

**Table 6:** Subgroup analysis of the discriminant power of the FAB-E to detect executive dysfunction between healthy controls and PD patients and between PD patients with (PD-CI) and without cognitive deficits (PD-nonCI) by sociodemographic characteristics.

	Control vs PD		PD-nonCI vs PD-CI	
	Optimal cutoff	AUC	Optimal cutoff	AUC
Base model	14.5	0.812	12.5	0.795
Including those less than 67 years old	13.5	0.648	15.5	0.636
Including those aged 67 years or older	14.5	0.819	12.5	0.778
Including only men	14.5	0.745	11.5	0.789
Including only women	14.5	0.788	12.5	0.828
Including only those with primary studies or less	14.5	0.794	11.5	0.700
Including only those with secondary studies or higher	15.5	0.724	12.5	0.837

Abbreviations: PD, Parkinson’s disease; PD-nonCI, Parkinson’s disease patients without cognitive impairment; PD-CI, Parkinson’s disease patients with cognitive impairment; AUC, area under the curve.

## Discussion

In this study, we have analyzed the psychometric properties of the Spanish version of the Frontal Assessment Battery (FAB-E) in a population with and without PD. The results showed good reliability, internal consistency, concurrent and discriminant validity, which indicates that the FAB-E may be a useful tool for evaluating EFs in PD patients. Moreover, the FAB-E may discriminate PD patients with CI and dementia and, therefore, could provide helpful information to follow the evolution of executive dysfunctions and to offer more comprehensive and targeted interventions for PD patients.

In line with previous studies examining the accuracy of the FAB as a valid tool for the assessment of executive functioning in PD patients[10–16], our results support the good performance of the Spanish version of the FAB provided by an extensive set of adequate measures of the psychometric capacity of the test. However, unlike these prior studies, we analyzed the reliability of the instrument as measured by ICC and test-retest correlations. Our findings showed a good degree of stability in the FAB-E scores over time, which suggests, along with the Conbrach’s alpha reliability coefficients, that the test items maintain a convincing consistency that produces valid and reliable outcome measures of executive functioning in PD patients.

Consistent with former studies, the results of the concurrent validity showed that the total FAB-E scores strongly correlated with the MMSE[10–14,16] and TMT[10,11,13] in PD patients. Specifically, the MMSE displayed a good relationship with the similarities and lexical fluency subtests of the FAB-E, which may be explained by the fact the MMSE evaluates language use and comprehension suggesting an overlap between the two measures. Regarding TMT, the both parts (i.e. TMT-A and -B) reflected the highest correlations in the motor series and go-no go FAB-E subtests, as it would be expected in people affected by PD. Thus, one possible explanation for the slightly poorer performance of the both TMT observed on the rest of the executive FAB-E domains could be likely attributed to the motor symptoms of PD[31], although it could be also partly accounted for by other factors such as age or educational level. The findings also indicated that the FAB-E showed solid correlations with the executive subtests of the RBT, which reinforces the ability of the FAB-E as valid tool for assessing executive dysfunction in PD patients. By contrast, only the prehension task of the EXIT-25 showed a fair correlation for the total score and for similarities and conflicting instructions scores of the FAB-E. Although the EXIT-25 is a short screening tool for assessing EFs as the FAB, it presents limitations due to the test may be sensitive to executive and non-executive functions[7], thereby indicating poor specificity[32]. Furthermore, as far as we know, the EXIT-25 has not previously used to assess EFs in PD patients, which makes it difficult to provide a suitable explanation for the unexpected estimates obtained.

An intriguing finding of our study was that the prehension behaviour FAB-E subtest suggested that response inhibition was the only EF that did not affect PD patients. Despite it is known that impaired inhibitory performance is a common deficit in PD[33], our results showed no differences in prehension FAB-E scores between healthy controls and PD patients. Similarly, the scores on this FAB-E subtest remained the same among PD patients, even accounting for their cognitive status. A tentative interpretation of that, according to the hypothesis by Palop et al.[34], could be supported by the fact that impaired cognitive function in neurodegenerative disorders can be compensated for additional processing, such as increased reliance on visual features detected in people with PD[35].

The growing recognition of the frequency and severity of cognitive deficits caused by PD has underlined the importance of its clinical implications as well as the need for a specific and targeted approach[28,36]. Accordingly, the interest in the diagnostic and screening power of neuropsychological testing in detecting cognitive decline in PD has also emerged as important research issue[13,36]. The discriminant capacity of the FAB as an accurate marker of differential diagnosis in several neurological disorders has been recently assessed[8]. Currently, the study by Biundo et al.[15] is the only work assessing the contribution of the FAB to the detect cognitive disorders in PD patients. Consistent with the ROC analysis of this previous study, our findings corroborate the adequate discriminant power of the FAB-E to distinguish CI and dementia in people with PD, although some dissimilarities between the studies are also evident. Due to different criteria used to define CI and dementia in PD, the optimal cutoffs were not similar; however, our estimates were lower. For comparative purposes, Biundo et al. included normative values for Italian population[9], i.e. <13.5 of the total FAB score, although the optimal values to detect CI (<15.1) or dementia (<13.7) in PD were higher than expected. Moreover, one important difference in our study is that we evaluated how the cutoffs obtained differed by sociodemographic features, since aspects such as age or educational level may have a strong influence on cognitive performance. In terms of clinical practice, it should be noted that our study adopted a more extensive approach providing helpful information for clinicians to make decisions at bedside. Apart from estimating the cutoffs to distinguish healthy subjects from people affected by PD, we explored which FAB-E subtests were the major contributors to a successful distinction between PD patients and healthy individuals. Moreover, to cover important lack of information on the changes of EFs in PD and to enhance the diagnostic accuracy of the instrument, we evaluated the discriminant power of the six FAB-E items according to the cognitive status of PD patients. Importantly, a detailed examination offered by the FAB-E scores could be also of relevance in advanced stages of disease in which executive dysfunction intensifies, given that the total FAB-E performance may play a role as marker of disease severity rather than a screening test[37].

This study presents several limitations. We did not use the Movement Disorder Society task force recommendation criteria to classify PD patients with CI or dementia. However, the MMSE is widely used as a standard bedside clinical test for cognitive dysfunction and, unlike other global cognitive tests, is insensitive to executive dysfunction. In addition, although the descriptive analysis indicated differences in the sociodemographic characteristics between the groups of the study participants, we examined their likely influence within the strata of age, sex, and educational level. Nevertheless, we are aware that the cut-off points, sensitivity and specificity values of the FAB-E are only applicable to PD patients with similar symptomatology as the participants in our study. EFs using the FAB-E should be also evaluated in heterogeneous samples of PD patients, addressing different stages of evolution of the disease, and also considering multiple levels of CI. Moreover, measuring these characteristics in different populations and diverse situations of illness would contribute to the improvement on the diagnostic accuracy and capacity of the FAB-E as a screening instrument.

In conclusion, the Spanish version of the FAB may be used as a reliable and valid screening tool to evaluate deficits in EFs in people with PD. Total and subtest scores of the FAB-E can provide a detailed assessment of frontal lobe functions and of the evaluation of cognitive decline in patients with PD, which may yield helpful information for clinicians and therapists during evaluation to guide their clinical decision making. Hence, the use of this instrument may be of help in cognitive preclinical diagnosis, improve the design of therapeutic interventions and complement the neuropsychological diagnosis of PD patients. For research purposes, it may be a valuable tool for researchers in epidemiological prospective studies conducted in PD patients.
